# Production and characterization of anti-*Campylobacter jejuni* IgY derived from egg yolks

**DOI:** 10.1186/s13028-017-0346-4

**Published:** 2017-12-06

**Authors:** Alexandre Thibodeau, Philippe Fravalo, Audrey Perron, Sylvette Laurent- Lewandowski, Ann Letellier

**Affiliations:** 1 Chaire de recherche industrielle du CRSNG en salubrité des viandes, Faculté de médecine vétérinaire, Université de Montréal, 3200 rue Sicotte, Saint-Hyacinthe, QUÉBEC Canada; 2 Centre de Recherche en Infectiologie Porcine et Avicole, Faculté de médecine vétérinaire, Université de Montréal, Saint-Hyacinthe, QUÉBEC Canada; 3 Groupe de recherche et d’enseignement en salubrité alimentaire, Faculté de médecine vétérinaire, Université de Montréal, Saint-Hyacinthe, QUÉBEC Canada

**Keywords:** Antibody, *Campylobacter jejuni* control, Chicken colonization, Egg yolk IgY, Immunoglobulin

## Abstract

**Background:**

*Campylobacter jejuni* is a major cause of foodborne disease having chickens as an important reservoir. Its control at the farm would lower the contamination of the final products and therefore also lower the risk of transmission to humans. At the farm, *C. jejuni* is rarely found in chickens before they reach 2 weeks of age. Past studies have shown that maternal antibodies could hamper *C. jejuni* gut colonization. The objective of this study was to compare protocols to use in order to produce anti-*C. jejuni* antibodies derived from egg yolks in the perspective to be used as feed additives for the control of chicken *C. jejuni* colonization. Laying hens were naturally contaminated with four well-characterized strains or injected with either outer membrane proteins or formalin-killed whole bacteria derived from these same strains. Eggs were collected and IgYs present in the yolks were extracted. The amount and the specificity of the recovered antibodies were characterized.

**Results:**

It was observed that injection yielded eggs with superior concentrations of both total and anti-*C. jejuni* antibodies. Equivalent performances for antibodies recovered from all protocols were observed for the ability of the antibodies to agglutinate the live *C. jejuni* homologous strains, to hinder their motility or to lyse the bacteria. Western blot analyses showed that proteins from all strains could be recognized by all IgY extracts. All these characteristics were strain specific. The characterization assays were also made for heterologous strains and weaker results were observed when compared to the homologous strains.

**Conclusions:**

Based on these results, only an IgY quantitative based selection can be made in regards to which protocol would give the best anti-*C. jejuni* IgY enriched egg-yolks as all tested protocols were equivalent in terms of the recovered antibody ability to recognized the tested *C. jejuni* strains.

## Background

*Campylobacter jejuni* is a Gram-negative bacterium that causes campylobacteriosis in humans. It is one of the leading bacterial foodborne pathogens worldwide [1]. Poultry meat products are a major source of *C. jejuni* for humans [2]. Up to 10^8^ *C. jejuni* colony forming units (CFU) per gram of caecal content can be found in chickens arriving at slaughter thus explaining the high rate of contaminated poultry products [3]. Lowering the level of caecal colonization may reduce the level of human exposure to this pathogen [2, 4]. To achieve this, on-farm strategies to prevent or at least to lower the bird’s intestinal colonization are required [5]. The transmission in a chicken flock is mainly horizontal [6]. Interestingly *C. jejuni* is usually detected in commercial chickens after the 2nd or 3rd week of age [1], which suggests the presence of natural barriers preventing *C. jejuni* early colonization. The presence of maternal antibodies is one mechanism that may explain that lag phase [5].

Maternal antibodies are transferred from the hens to the young poults via the egg by embryonic circulation [7, 8]. Maternal antibodies found in the young chicken can recognize *C. jejuni* [8–10] and limit intestinal colonization [9]. They can be detected in the first week of life, but afterwards they undergo a steady decline and are not detectable after 3 weeks of age [8]. Passively supplementing the chickens with antibodies, able to recognize *C. jejuni,* during the whole rearing period, could become an interesting approach to the control of colonization of chickens at the farm by this particular pathogen.

The egg can easily be used for production of such antibodies [11]. Eggs have previously been used for the production of antibodies destined to control the colonization of other pathogens with different rates of success. For example, uncharacterized egg powder from non-immunized hens showed the capacity to reduce colonization of *C. jejuni, Escherichia coli* and *Salmonella Typhimurium* [12]. Commercial egg powder derived from eggs collected from hens immunized with a live *Eimeria* vaccine showed a protection against a subsequent *Eimeria* challenge [13] while a similar approach directed against colonization of broilers by *Salmonella* sp. was unsuccessful [14]. For *C. jejuni*, different results have been achieved when using egg powder as a control strategy: a reduction of *C. jejuni* colonization [15] and no modification at all [16].

Almost all studies are concluding that a better characterization of the produced antibodies should be undertaken. Moreover, individually, these studies used different ways to increase the egg antibodies’ concentration and specificity. Therefore, it becomes crucial to evaluate different protocols in parallel to limit comparison bias brought by analysis of work done by multiple independent research teams. For *C. jejuni* specifically, several approaches can be used to immunize the laying hens and subsequently could be used to recover antibodies derived from the egg yolks. A simple approach is the natural colonization of the laying hens by *C. jejuni,* which increases the levels of antibodies in the recovered eggs [8, 9, 17]. On the other hands, whole *C. jejuni* proteins extracts [15–17], outer membrane proteins (OMP) [15, 17] and whole formalin-killed *C. jejuni* bacteria [17] have been used in chicken immunization trials.

The aim of this study was to compare different protocols for the production of egg yolks derived antibodies recognizing several strains of *C. jejuni*. Total and specific antibodies concentrations, the evaluation of their capacity to recognize *C. jejuni*, their capacity to block motility and to show bactericidal activity against live strains were assessed in the perspective of determining the most efficient method to produce IgYs that could eventually be used as feed additives for the on-farm control of chicken *C. jejuni* colonization.

## Methods

### Strains

Strains (*C. jejuni* A2008A, *C. jejuni* B2008A, *C. jejuni* G2008B, *C. jejuni* RM1221), previously characterized by our laboratory [18–21], were used and are referred in this study as the homologous strains. Strains A2008A and G2008B are hyper-competitive for chicken colonization while strain B2008A is a poorer competitor. Strain RM1221, isolated from a case of poultry associated campylobacteriosis, is a fully sequenced strain [22]. Control strains 81-176 [23], 81116 [24], ATCC 700819 alias NCTC11168 [25] and ATCC 33291 were used as heterologous strains.

### Experimental design

All experiments were performed with the approval of the Ethics Committee (CEUA) of the Faculty of Veterinary Medicine (FMV) of the University of Montreal Canada (FMV), approval #Rech-1740. Forty specific pathogen free (SPF) White Leghorn hens acquired from the Canadian Food Inspection Agency (Ottawa, ON, Canada), of 5 weeks of age and tested negative by cloacal swabs for *C. jejuni*, were raised in a level two biosecurity housing facility in FMV.

During the whole study, hens were placed in individual cages and wing-tagged. All birds had access to water and were fed ad libitum. Hens received a standard commercial feed, bought from a local feed mill (Aliment Natur-Aile, COOP Fédérée, Canada) composed of 17% protein, 3.5% calcium, 3% fat, 5% fiber and 0.3 mg/kg selenium. Feed was tested by culture for the absence of *C. jejuni*. Upon arrival, the 40 laying hens were divided into two groups: 10 laying hens were placed in room #1 (for inoculation with *C. jejuni*) and 30 laying hens, housed in an independent room (room #2), were further distributed in 3 groups of 10 hens.

From day 1 to the end of the study, fresh caecal droppings were weakly tested to confirm the absence or presence of *C. jejuni*. At 16 weeks of age, the 10 hens of room #1 were orally inoculated with a 1 mL suspension containing 3.6 × 10^5^ CFU of a mix of four different *C. jejuni* strains (GR2-INO). Also at 16 weeks of age, 10 hens from the room #2 were injected subcutaneously with 100 µg of *C. jejuni* OMP extracts solubilized in 1 mL of HEPES buffer (Fisher Scientific, Ottawa, ON, Canada) containing 50% of Freund’s incomplete adjuvant (Sigma-Aldrich Corporation, St. Louis, MO, USA) (GR3-OMP). Ten other hens were injected subcutaneously with 10^9^ formalin-killed whole *C. jejuni* suspended a 1 mL of HEPES buffer containing 50% of Freund’s incomplete adjuvant (GR4-BACT). The antigens prepared for these injections originated from the same equivalent mix of the four strains used for the oral inoculation. The 10 remaining hens were the control group: 5 hens received an oral dose of 1 mL of tryptone salt (TS) solution (Innovation Diagnostic Inc., Montreal, QC, Canada) (GR1-CINO) or were injected with 1 mL of HEPES buffer (Fisher Scientific) containing 50% of adjuvant (GR1-CIM). These two hens group were considered being the negative control group (GR1-CTL). Injection boosters were given at 20 and 28 weeks of age (week 4 and 12 post-injection). Starting with the first egg laid and until the end of the experiment, all eggs were collected daily, identified and stored at 4 °C for egg yolks IgY extraction.

### Oral inoculating suspension

Briefly, − 80 °C frozen aliquots of each *C. jejuni* strains were cultured on *Campylobacter* Blood Free Selective Medium (mCCDA) (Innovation Diagnostic Inc.) for 24 h, at 42 °C, in a microaerobic atmosphere, using Campy*Gen* gas pack (Oxoïd, Nepean, ON, Canada). Strains were then transferred onto tryptic soy Agar (TSA) containing 5% (v/v) defibrinated sheep blood (Fisher Scientific). Each strain was suspended in 1 mL of TS (Innovation Diagnostic Inc.) to reach an optic density (630 nm) of 1.0 and subsequently diluted to obtain approximately 10^5^ CFU/mL. The four strains were then mixed in equal volumes and 1 mL of this suspension was used to orally inoculate the GR2-INO chickens. All suspensions were enumerated by culture on Brucella Agar (Innovation Diagnostic Inc.) after 48 h incubation, at 42 °C, in a microaerobic atmosphere.

### Total protein extraction

Total protein extracts were obtained by a sonication method described by de Melo et al. [26] with minor changes. *C. jejuni* strains from an overnight culture on TSA blood agar were harvested and suspended in HEPES buffer (Fischer Scientific). The suspension was sonicated on ice five times (30 s each) with a 1 min cooldown period between each burst. Cell debris were removed by centrifugation at 10,000×*g* at 4 °C for 10 min. The total proteins were recovered in the supernatants and stored at – 20 °C until used.

### OMP extractions

OMP of the homologous *C. jejuni strains* were obtained based the *N*-lauryl sarcosyl method described by Hobbs et al. [11]. After lysis, the membranes were collected by centrifugation at 100,000×*g* for 1 h at 4 °C. The pellet was resuspended in 2 mL of 10 mM HEPES buffer (pH 7.4) and centrifuged again for 1 h at 100,000×*g*. The resulting pellet was resuspended in 10 mM HEPES (pH 7.4) buffer. The protein concentration was determined by measuring the absorbance at 280 nm using a NanoDrop ND-1000. *N*-lauryl sarcosine (Sigma-Aldrich Cooperation, St. Louis, MO, USA) was added to the sample at a protein-to-detergent ratio of 1:4 (wt/wt) and incubated at 37 °C for 30 min with shaking. The sarkosyl-treated membranes were then centrifuged at 100,000×*g* for 1 h at 4 °C. The pellet was washed with 10 mL of 10 mM HEPES (pH 7.4) and centrifuged again. The pellet was resuspended in 500 µL of a 10 mM HEPES (pH 7.4), buffer. The OMP extracts were kept at – 20 °C.

### Formalin-killed Campylobacter

Each strain was suspended in 1 mL TS solution (Innovation Diagnostic Inc.) to obtain an absorbance of 1.0 measured at 630 nm, corresponding to approximately 10^9^ CFU/mL. Formalin (Sigma-Aldrich Corporation) was then added to reach a final percentage of 1%. The mixture was incubated at 4 °C for 24 h. Cells were washed four times, centrifuged at 4000x*g* for 10 min at 4 °C and washed with 10 mM HEPES buffer. The resulting suspension was inoculated on mCCDA (Innovation Diagnostic Inc.) for 48 h, at 42 °C, in a microaerobic atmosphere to confirm that the bacteria were no longer cultivable. Microscopic observations were also done to confirm that the cellular morphology was retained.

### Antibodies extraction

A chloroform-based method described by Polson [27] with slight modification was used for the immunoglobulin extraction. After the separation from the egg albumen, the yolk was poured into a 50 mL conical tube. Twice the yolk volume of Dulbecco’s PBS (Sigma-Aldrich Corporation.) was added and the samples were mixed thoroughly by vortex. An equal volume of chloroform (Sigma-Aldrich Corporation) was added to the yolk and PBS mixture, and the contents were mixed vigorously to produce a thick emulsion. After centrifugation (16,300x*g*, 20 min, room temperature), the aqueous phase containing the IgY was collected, aliquoted, and stored at − 20 °C until the analysis.

### Enzyme-linked immunosorbent assay

The levels of total and anti-*Campylobacter* IgYs in egg yolks were determined using Chicken IgG ELISA Quantitation Set (Bethyl Laboratories, Montgomery, TX, USA) following the manufacturer’s instruction. The working concentrations for the determination of the total IgY were 1:20,000 for GR2-INO and GR1-CTL while 1:10,000 was selected for the other groups.

The sample working dilution for the titration of the *anti*-*Campylobacter* antibodies was 1:75 for groups GR2-INO and GR1-CTL. A working dilution of 1:500 for groups GR3-OMP and GR4-BACT was retained. The plates were coated with 2 μg/mL of total protein extracted from an equal mix of the four *C. jejuni* homologous strains instead of the goat anti-chicken IgG (IgY)-Fc fragment antibody.

### Agglutination assays

A bacterial suspension (OD 630 nm of 1.5) of each strain (homologous and heterologous) was obtained in a NaCl solution (0.85%, w/v) from an overnight culture grown on TSA containing 5% defibrinated sheep blood. In an agglutination plate, one drop of each suspension was added to one drop of each non-diluted IgY extracts or a drop of PBS. Agglutination was recorded within 2 min incubation at room temperature.

### Immunoblot

Protein samples and molecular weight markers (Amersham High-Range Rainbow Molecular Weight Marker, GE Healthcare Bio-Sciences Corporation, Piscataway, NJ, USA) were diluted in sample buffer (0.5 M Tris (pH 6.8), 2% sodium dodecyl sulfate (SDS), 10% glycerol, 5% mercaptoethanol). Total protein (75 µg) of each *C. jejuni* strain was loaded on a 10% *Bis*-Acrylamide (Fisher Scientific) separating gel. Gels were run at 4 °C in the running buffer (25 mM Tris, 0.2 M glycine, 0.1% SDS) at 16 mA during 16 h. After SDS-PAGE, proteins were transferred to a PVDF membrane (Bio-Rad Laboratories, Mississauga, ON, Canada) using the transfer buffer (0.125 M Tris-base, 0.1 M glycine) at 100 V for 3 h at 4 °C.

The membranes were blocked in *Tris*-buffered saline (TBS) supplemented with 2% (wt/vol) skim milk powder (Smucker Foods of Canada Co. Markham, ON, Canada), for 1 h at room temperature and thereafter incubated 2 h with the different IgY extracts added to the blocking buffer. The dilutions of the IgY extracts were 1:50 for GR1-CTL and GR2-INO or 1:250 for the other groups. After five washes in TBS, the blots were incubated 1 h at room temperature with a goat anti-chicken IgG (IgY)-Fc fragment antibody HRP conjugated (Bethyl Laboratories) at a concentration of 1:3000. After being washed five times, the blots were developed by incubation in a solution containing H_2_O_2_ (Fisher Scientific), methanol (Fisher Scientific) and 4-chloro-1-naphtol (Sigma-Aldrich Corporation) for 20 min.

### Motility assay

Motility assays were performed as described previously [3] with slight modifications. A – 80 °C frozen aliquot of each strain was cultured onto TSA containing 5% (v/v) defibrinated sheep’s blood (Fisher Scientific). Each strain was suspended in 1 mL of TS (Innovation Diagnostic Inc.) to obtain an optic density (630 nm) of 0.18. Bacterial suspensions (1 µL) were then added to 100 µL of IgY extracts or 100 µL of PBS. The bacterial suspensions were incubated for 30 min and 10 µL was spotted onto the surface of a *Brucella* plate containing 0.4% agar. Motility plates were incubated for approximately 40 h at 37 °C under microaerobic conditions. The growth diameter (mm) was then measured. The experiment was repeated three times.

### Bactericidal assay

The bactericidal assay was performed according to Sahin et al. [9]. Briefly, each test tubes contained 50 µL of the bacterial suspension, 50 µL of 1:5 (in PBS) complement (sera recovered from *C. jejuni* negative chickens) and 10 µL of antibody extracts or PBS. Control tubes included (i) bacteria + complement only; (ii) bacteria + antibody only; and (iii) bacteria + PBS only. After 1 h incubation at 37 °C, 100 µL of the bacterial suspension was enumerated onto *Brucella* agar plates (Innovation Diagnostic Inc.) incubated for 48 h, at 42 °C, in a microaerobic atmosphere. The percentage of reduction in the number of live *C. jejuni* was calculated by the following formula: [CFU (bacteria + complement only)—CFU (bacteria + antibody + complement)]/CFU (bacteria + complement only) × 100. The assay was repeated three times.

### Statistical analysis

All statistical analyses were computed in GraphPad v6 (Prism, LaJolla, USA). An alpha value of 0.05 was chosen as the significance level. When more than two groups were compared, the Kruskal–Wallis test was used while two-by-two comparisons were analyzed with the Mann–Whitney test.

## Results

### Egg yolks total and anti-*C. jejuni* IgYs

**Fig. 1:**
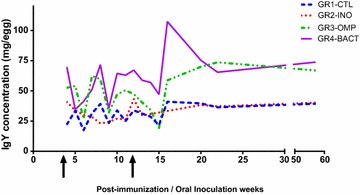
Quantification by ELISA of total IgY found in the egg yolks, post-injection of the laying hens. Each result represents the mean IgY concentrations in extracts from each laying hens group. GR2-INO (orally inoculated), GR3-OMP (injected with OMP), GR4-BACT (injected with formalin-killed whole bacteria), GR1-CTL (control groups); The arrows on the x-axis mark the boosters

Total IgY per yolk increased in time to reach a plateau at 22 weeks post-immunization (Fig. 1). A drastic increase could be seen after each reinjection. GR3-OMP and GR4-BACT eggs were the ones with the highest IgY content while GR2-INO hen’s layed eggs had similar total IgY levels as the eggs collected in group GR1-CTL.

**Fig. 2:**
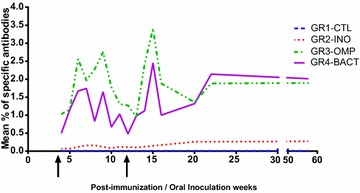
Quantification by ELISA of *C. jejuni* specific IgY found in the egg yolks, post-injection of the laying hens. Each result represents the mean IgY concentrations in extracts from each laying hens group. GR2-INO (orally inoculated), GR3-OMP (injected with OMP), GR4-BACT (injected with formalin-killed whole bacteria), GR1-CTL (control groups); The arrows on the x-axis mark the boosters

The concentration of *C. jejuni* specific IgYs increased with time and was drastically improved by the boosters that were given 4 and 12 weeks after the first injection (Fig. 2), for GR3-OMP and GR4-BACT eggs. At 22 weeks post-injection, a maximal level of anti-*C jejuni* IgY was reached and remained stable until the end of the experiment. At 22 weeks, groups GR3-OMP and GR4-BACT produced significantly higher (P < 0.05 Mann–Whitney) proportion of specific IgY than group GR2-INO (1.9% of specific antibodies for GR3-OMP and GR4-BACT compared to 0.3% for GR2-INO). No *C. jejuni* specific IgY could be detected in the group GR1-CTL.

### Immunoblot protein recognition patterns

**Fig. 3:**
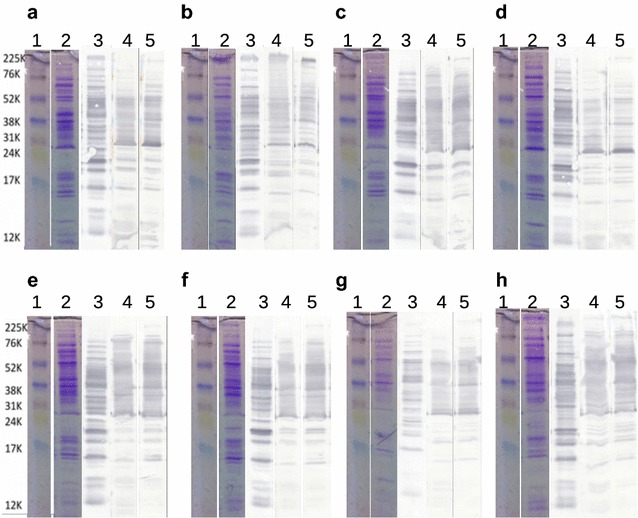
Immunoblot analysis of egg-yolks derived IgY recognition profiles of total proteins of different *C. jejuni* strains. No proteins were recognized by group GR1-CTL egg yolks extracts. Lane 1: ladder, Lane 2: total proteins of the strain tested stained with Coomassie blue; Lanes 3 to 5, respectively: proteins recognized by the IgY extract from GR2-INO (orally inoculated), GR3-OMP (OMP) and GR4-BACT (formalin-killed whole bacteria); (**a**–**d**) Homologous *C. jejuni* strains: A2008A, B2008A, G2008B, RM 1221; (**e–h**) = Heterologous *C. jejuni* strains: 81116, 81-176, ATCC 700819, ATCC 33291

Antibodies recovered from GR1-CTL did not recognize any proteins from tested strains (data not shown). Antibodies recovered from the egg yolks of groups GR2-INO, GR3-OMP and GR4-BACT were able to recognize proteins from both homologous and heterologous strains (Fig. 3). The *C. jejuni* total protein recognition pattern for GR2-INO derived antibodies was different from the ones observed for GR3-OMP and GR4-BACT, while recognition patterns from both immunized groups looked similar. Moreover, this comparison established that recognition patterns appeared strain-dependant.

### Agglutination

No agglutination reaction could be observed for group GR1-CTL yolk extracts while a positive reaction toward the homologous strains was noted for all other extracts (Table 1). With the exception of strain 81116 for GR2-INO extracts, no agglutination was noted for the heterologous strains.

**Table 1:** Agglutination reaction after contact of each IgY extract with a suspension of different *C. jejuni* strain

*C. jejuni* strain	GR1-CTL IgY extract	GR2-INO IgY extract	GR3-OMP IgY extract	GR4-BACT IgY extract
A2008A	None	Positive	Positive	Positive
B2008a	None	Positive	Positive	Positive
G2008B	None	Positive	Positive	Positive
RM1221	None	Positive	Positive	Positive
81116	None	Positive	None	None
81-176	None	None	None	None
ATCC 700819	None	None	None	None
ATCC 33291	None	None	None	None

None = no observed reaction; Positive = observed reaction

Homologous *C. jejuni* strains: A2008A, B2008A, G2008B, RM 1221

Heterologous *C. jejuni* strains: 81116, 81-176, ATCC 700819, ATCC 33291

GR2-INO (orally inoculated), GR3-OMP (immunized with OMP), GR4-BACT (immunized with formalin-killed whole bacteria), GR1-CTL (control group)

### Motility and bactericidal assays

For the motility assay, no difference was observed. On the other hand, the bactericidal assay clearly demonstrated a loss of cultivability of *C. jejuni* associated with the extracts from GR2-INO, GR3-OMP and GR4-BACT (Table 2). The mean cultivability reduction of all the homologous strains tested was higher for all groups compared to the group GR1-CTL. The same result was observed for two of the heterologous strains: *C. jejuni* ATCC 700819 and *C. jejuni* ATCC 33291. The mean percentage reductions were overall higher for the homologous strains than the heterologous strains. Only strain RM1221 showed a difference in the IgY bactericidal activity depending on the chicken group. In this case, GR3-OMP and GR4-BACT IgY extracts induced a higher bactericidal effect than the extracts from group GR2-INO.

**Table 2:** Bactericidal efficiency of IgY extracts

Strain	GR1-CTL Reduction	GR2-INO Reduction	GR3-OMP Reduction	GR4-BACT Reduction
Homologous				
A2008A	17.35^a^ (6.53)	57.57^b^ (3.39)	50.30^b^ (4.39)	53.20^b^ (17.13)
B2008A	29.57^a^ (12.60)	95.12^bc^ (5.50)	60.45^bd^ (17.78)	68.20^bd^ (8.05)
G2008B	21.50^a^ (14.73)	99.61^bc^ (0.44)	48.24^bd^ (26.87)	80.08^bd^ (13.22)
RM 1221	22.34^a^ (12.52)	49.71^c^ (20.42)	84.11^bd^ (9.79)	64.75^b^ (20.98)
Mean homologous	22.62^a^ (12.41)	79.92^bc^ (24.67)	60.03^bd^ (21.76)	67.35^b^ (17.21)
Heterologous				
81116	34.92 (21.48)	39.07 (22.01)	33.34 (8.97)	33.46 (16.54)
81-176	34.92 (21.48)	39.07 (22.01)	33.34 (8.97)	33.46 (16.54)
ATCC 700819	14.42^a^ (5.04)	37.10^b^ (7.76)	42.89^b^ (33.36)	46.31^b^ (3.08)
ATCC 33291	6.54^a^ (5.17)	26.30^b^ (10.00)	31.53^b^ (12.54)	28.76^b^ (10.96)
Mean heterologous	20.95^a^ (18.58)	34.86^b^ (14.78)	35.55^b^ (9.65)	35.79^b^ (12.88)

Mean reduction percentage of the initial count for the homologous and heterologous strains

() the standard deviation for 3 biological replicates

Homologous *C. jejuni* strains: A2008A, B2008A, G2008B, RM 1221

Heterologous *C. jejuni* strains: 81116 and ATCC 33291

On a same row a different than b and c different than d, P < 0.05, Kruskal–Wallis test followed by pairs of Mann–Whitney tests

GR2-INO (orally inoculated), GR3-OMP (immunized with OMP), GR4-BACT (immunized with formalin-killed whole bacteria), GR1-CTL (control group)

## Discussion

Different protocols designed to induce a strong anti-*C. jejuni* IgY response in the egg yolks of laying hens were compared based on the fine characterization of the egg yolks antibody content. This study also used different strains as source material for the induction of the specific anti-*C. jejuni* IgY in an attempt to allow the produced IgY to recognize a wide array of strains. Specific antibodies against *C. jejuni* were produced after an oral inoculation of live *C. jejuni* (mimicking natural colonization) or after an injection with *C. jejuni* OMP or formalin-killed whole bacteria.

This study is reporting the levels of antibody found in term of concentrations while most studies are reporting titers [15] or optic densities [8, 9, 14, 16]. Reporting the actual concentration of IgY is more practical to compare different studies. In our study, there were strong differences in IgY concentration depending on the chicken groups, as the oral inoculation stimulated a lesser IgY production compared to GR3-OMP and GR4-BACT.

Immunoblots showed that an oral inoculation or both injections (OMP or formalin-killed *C. jejuni*) induced the production of antibodies able to recognize a large pattern of proteins from both homologous and heterologous strains (Fig. 3). Different recognition patterns by IgY extracts for different *C. jejuni* strains were also observed by previous studies [8, 10].

The IgY extracts were also able to recognize and agglutinate the live homologous strains. The addition of the different IgY extracts failed to induce agglutination of most heterologous strains. Since the immunoblot results confirmed the recognition of both homologous and heterologous strains by the antibodies in a strain dependant manner (Fig. 3) this difference in agglutination between homologous and heterologous strains could indicate greater antibody avidity for homologous strains or that the recognized heterologous strain proteins do not offer binding sites that promote agglutination.

The IgY extracts were also tested for their ability to hinder *C. jejuni* motility as it was demonstrated that motility is required for the full colonization of chickens [28]. In this experiment conditions, none of the IgY extracts were able to block or reduce the motility of *C. jejuni* despite the fact that whole bacteria can be agglutinated by these same IgY extracts. In a previous study [10], it was showed that a serum with antibodies against *C. jejuni* was able to reduce the motility of *C. jejuni* homologous strains. On the other hand, in a recent study, IgY derived from hyperimmune egg yolks were not able to reduce *C. jejuni* motility but were able to affect *C. jejuni* colonization of chickens [15] thus illustrating a great discrepancy between published results.

The second in vitro characterization assay on live bacteria was the analysis of the bactericidal effect of the IgY extracts. The results showed a diminution of the number of bacteria after the co-incubation of the antibodies and the complement, which suggests the capacity of the specific IgYs to promote lysis of the homologous as well as some heterologous strains. Bactericidal assays against *C. jejuni* were performed by other research groups using sera as a source of antibody against *C. jejuni*. Maternal antibodies contained in a 2-day-old chick sera were able to reduce the CFU count for homologous but not heterologous strains tested [9] while day-old chick sera reduced the CFU count of two heterologous strains with a higher reduction for one strain than the other [8]. Our results also showed that the intensity of the bactericidal effect is strain dependent and could not be related to a specific IgY production protocol.

In this study, all evaluated protocols yielded egg yolk derived IgY that were able to recognize the tested *C. jejuni* strains, with best results achieved against the homologous strains compared to the heterologous ones. This illustrates that in vivo trials aimed at determining the efficiency of hyperimmune egg powders for the control of *C. jejuni* chicken colonization should include both homologous and heterologous strains as chickens raised on commercial farms are likely to be exposed to numerous *C. jejuni* strains.

Higher concentration of IgY could be recovered in extracts derived from GR3-OMP and GR4-BACT eggs. On the other hand, when comparing the different extracts ability to recognize *C. jejuni*, it is surprising that the antibodies obtained from colonized hens, that mimic the production of maternal antibodies, gave results similar to the other two groups despite a lower concentration of both total and anti-*C. jejuni* IgYs in the egg yolks. It can be hypothesized that the in vivo colonization process induced the expression of colonization factors different than what is produced in vitro [29], creating an immune response more efficient to recognize *C. jejuni*. This clearly illustrates that quantity alone is insufficient to assess the effectiveness of a given protocol to produce IgY enriched egg yolks for an eventual use to block colonization of food animals by the foodborne pathogen *C. jejuni*. It also illustrates the need for a better understanding of *C. jejuni* proteome expressed during colonization in order to stimulate in vitro the production of proteins more relevant to colonization which would induce a much better production of antibody by the chickens.

## Conclusions

The inoculation of OMP or bacterine induced the greatest concentration of anti-*C. jejuni* IgYs in the corresponding chicken egg yolks but on the other hand, all protocols, including natural colonization, yielded equivalent results when the recovered antibodies were further characterized. This is needed to be taken into account when trying to select for the best protocols to use for the further development of egg-yolk derived antibody as an in-feed control strategy for *C. jejuni* chicken colonization.

## Abbreviations

OMP: outer membrane proteins
CEUA: ethics committee if the faculty of veterinary medicine
FMV: Faculty of Veterinary Medicine
SPF: specific pathogen free, i.e. free from any chicken infection as well as *Salmonella* and *Campylobacter*
TS: tryptone salt
mCCDA: *Campylobacter* Blood Free Selective Medium
TSA: tryptic soy Agar
ELISA: enzyme-linked immunosorbent assay
TBS: tris-buffered saline
